# Laparoscopic hysterectomy with intraoperative treatment of vaginal strictures: A case report

**DOI:** 10.1016/j.crwh.2025.e00733

**Published:** 2025-07-14

**Authors:** Maddison McLellan, Antoinette Abdelmalek, Nkiruka Chuba

**Affiliations:** a Stanford University, Department of Orthopaedic Surgery, 500 Pasteur Rd, Stanford, CA 94304, United States of America; b University of California Irvine, Department of Obstetrics and Gynecology, 1001 Health Sciences Rd, Irvine, CA 92617, United States of America

**Keywords:** Vaginal stricture, Endometriosis, Laparoscopic hysterectomy

## Abstract

There is limited data regarding management of vaginal strictures with a concurrent hysterectomy, in all realms, including pre-, intra- and postoperative care. A 44-year-old nulligravid woman with multiple vaginal strictures and clinically suspected endometriosis was evaluated preoperatively and underwent laparoscopic hysterectomy with simultaneous dilation of vaginal strictures without complication. Postoperative care included vaginal estrogens and vaginal dilators. Concurrent vaginal stricture dilation and laparoscopic hysterectomy can be successful and without complication with the use of intraoperative dilation and postoperative estrogens.

## Highlights

• seemingly the first case of concurrent laparoscopic hysterectomy and intraoperative vaginal stricture dilation in a patient with multiple strictures and suspected endometriosis.
• Adaptive surgical techniques enabled successful hysterectomy despite the cervix inaccessability.
• Postoperative vaginal estrogen maintained structure patency in a non-sexually active patient.
• Pathology correlation confirmed endometriosis and benign ovarian cystadenofibromas, supporting clinical diagnosis.
• Demonstrates a feasible approach for complex cases, though long-term outcomes require further study.

## 1 Introduction

There is limited data regarding the pre-, intra- and postoperative management of vaginal strictures during concurrent hysterectomy. [[1], [2], [3]] Given the limited access to the vaginal canal/cervix, strictures pose a challenge to laparoscopic hysterectomy; a vaginal hysterectomy may not be possible. [4] Potential surgical strategies included addressing the strictures perioperatively or intraoperatively alongside a hysterectomy. [3,5] Treatment for the strictures, if deferred to operative management, include serial dilation/manipulation under anesthesia, or lysis of adhesions and placement of stents, dilators, or mesh. [5] The Veccheitti procedure has been successful, though not studied with hysterectomy. [1] For multiple strictures, surgical techniques include treating/correcting a single stricture by direct incision or performing a Zplasty. [3] A laparoscopic approach may be used for hysterectomy, but alternatives for uterine manipulation may be needed. [6,7]

## 2 Case Presentation

A 44-year-old nulligravid woman with concern for endometriosis and enlarged complex cysts of bilateral ovaries presented for total laparoscopic hysterectomy and bilateral salpingo-oophorectomy due to a history of abnormal uterine bleeding and dysmenorrhea for one year. She had been sexually active with men until five years prior to consultation but had no plans to become sexually active again after surgery. She also had no known history of sexually transmitted infections (STIs), dyspareunia or tampon use.

**Fig. 1:**
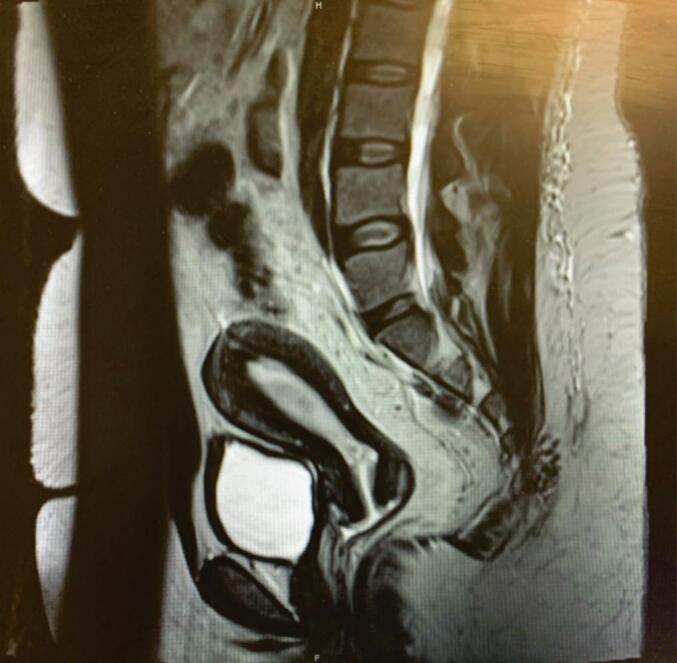
Vaginal Strictures visualized on MRI pelvis with and without contrast.

Magnetic resonance imaging of the pelvis with and without contrast preoperatively revealed a single 1.0 cm fibroid (FIGO 6), a right ovarian multiloculated cystic lesion measuring 8.1 × 13.8 × 9.6 cm, a left ovarian multiloculated cystic lesion measuring 3.7 × 5.3 × 5.2 cm with questionable thick septations without definite enhancing solid tissue and a normal-sized uterus (Fig. 1). Earlier transvaginal ultrasound had revealed similar findings with no evidence of endometrial abnormality.

Pre-operative pelvic examinations revealed vagina shortened to 4 cm with no visible cervix; exam was limited due to vaginal length. A blind pap smear was collected as the speculum could not be advanced. The bimanual exam revealed no palpable cervical tissue and was limited by shortened vagina. Pap smear returned negative for intraepithelial lesions and malignancy, and negative for high-risk HPV. While the cause of the vaginal strictures was unclear, the patient noted a history of trauma to her pelvic area as a child, though medical evaluation at the time per patient was normal. The healing process after injury may be a reason for stricture formation; however, the exact injury and its severity, along with management, were unclear and no medical records were available. The patient had no history of systemic illness or disease processes that would contribute to stricture formation and no concerning findings throughout the rest of her body to suggest abnormal healing or disease. Her last menstrual period had been 12 days before the operation described here.

On the day of the operation, the pelvic examination under anesthesia revealed vaginal stenosis approximately 4 cm up the vaginal canal. Two narrow canals were visualized, within the stenotic area at the midline and on the right. The midline canal was pinpoint, and a uterine sound was unable to pass. The left-sided canal was 0.5 cm in diameter and underwent serial dilation. The cervix was not palpated beyond the stenotic region.

At the start of the hysterectomy, a uterine manipulator could not be placed, so after placement of a 5 mm camera and accessory ports, a 12 mm port was placed suprapubically under direct visualization. The uterine fundus was injected with dilute vasopressin and a laparoscopic myoma screw was placed at the fundus for uterine manipulation. With adequate uterine manipulation and improved visualization, the bilateral adnexa and fallopian tubes were successfully separated and transected as well as the bilateral round and IP ligaments and bladder flaps. The bilateral uterine and utero-ovarian arteries were skeletonized, cauterized, and cut with ligature devices. The bladder was resected off the lower uterine segments, and two areas of likely endometriosis were transected from the bladder peritoneum to be sent to pathology. The ureters were identified transperitoneally and bilaterally. After the uterine arteries were skeletonized, cauterized and cut with the Sonicscision, the colpotomy was created utilizing an initial drilling technique to incise the anterior vaginal fornix to identify the cervix. The cervix was grasped and everted, with care to hug the cervix as the colpotomy was completed circumferentially and subsequently closed laparoscopically with 2–0 V-lock suture in running fashion. The area was irrigated, and Surgiflow was placed along the vaginal cuff, noted to be hemostatic. The uterus, cervix, bilateral ovaries, and fallopian tubes were removed via contained morcellation via endocatch bag.

Under laparoscopic visualization, the sites of vaginal stenoses were mechanically dilated up to size 61 French using lacrimal duct dilators as vaginal manipulators could not be used. There was no communication between the vagina and vaginal cleft. A small bridge between the two dilated canals was identified, transected and resected with a Bovie cautery via a vaginal approach. The stenosis was improved to 3 cm diameter vagina. The vagina was packed with Premarin soaked gauze and the foley was replaced.

Pathology reports revealed endometriosis on the left bladder peritoneum, bilateral ovarian benign papillary serious cyst adenofibromas, leiomyomas on the uterus, as well as normal cervical and fallopian tube pathology. Foley catheter and vaginal packing were removed, and patient was discharged home on postoperative day 1. She was prescribed 0.0625 mg/g topical estrogen cream twice weekly.

The patient recovered well postoperatively. Her postoperative pelvic exams revealed vaginal strictures, with an inability to pass the speculum through. On bimanual exam, her stricture was noted to be softer than preoperatively, and her vaginal cuff was palpable through the stricture and noted to be intact. Two weeks after the operation, the patient's stricture was noted to be 1.5 cm in diameter. Two months post-operation, the patient's stricture was 2 cm in diameter. Her pain was well controlled, and she was managed with vaginal dilators and topical estrogen therapy post-operatively. The patient was thereafter lost to follow-up and so it is not possible to extend the immediate postoperative recommendations.

## 3 Discussion

The present case suggests that a limited preoperative examination does not limit the operative and postoperative care of vaginal strictures as laparoscopic hysterectomy was able to be performed successfully and without complication in conjunction with manual dilation of two vaginal strictures under anesthesia. For patients who are being assessed, undergoing and/or recovering from gynecological surgery with co-existing vaginal strictures, the laparoscopic approach is possible and can be successful, though it may require additional preoperative work-up, intraoperative alternative methods and more extensive routine postoperative care.

## Contributors

Maddison McLellan contributed to patient care, acquiring and interpreting the data, drafting the manuscript, undertaking the literature review and revising the article critically for important intellectual content.

Antoinette Abdelmalek contributed to revising the article critically for important intellectual content.

Nkiruka Chuba contributed to patient care, acquiring and interpreting the data, and revising the article critically for important intellectual content.

All authors approved the final submitted manuscript.

## Patient consent

Written informed consent was obtained from the patient for publication of this case report.

## Provenance and peer review

This article was not commissioned and was peer reviewed.

## Funding

No funding from an external source supported the publication of this case report.

## Declaration of competing interest

The authors declare that they have no conflict of interest regarding the publication of this case report.
